# Chromosomally Located *fosA7* in *Salmonella* Isolates From China

**DOI:** 10.3389/fmicb.2021.781306

**Published:** 2021-12-28

**Authors:** Jing Wang, Yan Wang, Zhen-Yu Wang, Han Wu, Cai-Yue Mei, Peng-Cheng Shen, Zhi-Ming Pan, Xinan Jiao

**Affiliations:** ^1^Key Laboratory of Prevention and Control of Biological Hazard Factors (Animal Origin) for Agrifood Safety and Quality, Ministry of Agriculture of China, Yangzhou University, Yangzhou, China; ^2^Jiangsu Key Laboratory of Zoonosis, Jiangsu Co-innovation Center for Prevention and Control of Important Animal Infectious Diseases and Zoonoses, Yangzhou University, Yangzhou, China; ^3^Joint International Research Laboratory of Agriculture and Agri-Product Safety, Yangzhou University, Yangzhou, China

**Keywords:** fosfomycin resistance, *fosA7*, *Salmonella* Derby, chromosome-located, ST40

## Abstract

This study aimed to investigate the prevalence of fosfomycin *fosA7* in *Salmonella enterica* isolates from food animals and retail meat products in China and the impact of *fosA7* on bacterial fitness. A total of 360 *Salmonella* isolates collected from 11 provinces and cities in China were detected for *fosA7*. All *fosA7*-positive *Salmonella* isolates were determined minimum inhibitory concentrations (MICs) and sequenced by Illumina Hiseq. The *fosA7* gene of *S*. Derby isolate HA2-WA5 was knocked out. The full length of *fosA7* was cloned into vector pBR322 and then transformed into various hosts. MICs of fosfomycin, growth curves, stability, and fitness of *fosA7* were evaluated. The *fosA7* gene was identified in *S*. Derby (ST40, *n* = 30) and *S*. Reading (ST1628, *n* = 5). MICs to fosfomycin of 35 *fosA7*-positive isolates were 1 to 32 mg/L. All *fosA7* were located on chromosomes of *Salmonella*. The deletion of *fosA7* in HA2-WA5 decreased fosfomycin MIC by 16-fold and slightly affected its fitness. The acquisition of plasmid-borne *fosA7* enhanced MICs of fosfomycin in *Salmonella* (1,024-fold) and *Escherichia coli* (16-fold). The recombinant plasmid pBR322-*fosA7* was stable in *Salmonella* Typhimurium, *S*. Pullorum, *S*. Derby, and *E. coli*, except for *Salmonella* Enteritidis, and barely affected on the growth of them but significantly increased biological fitness in *Salmonella*. The spread of specific *Salmonella* serovars such as *S*. Derby ST40 will facilitate the dissemination of *fosA7*. *fosA7* can confer high-level fosfomycin resistance and enhance bacterial fitness in *Salmonella* if transferred on plasmids; thus, it has the potential to be a reservoir of the mobilized fosfomycin resistance gene.

## Introduction

In recent decades, multidrug-resistant and extensively drug-resistant Gram-negative pathogens are increasingly reported worldwide, posing a serious threat to clinical medicine (Karaiskos et al., 2019). Available antibiotics are very limited to be used in clinical practice; thus, older antibiotics such as colistin and fosfomycin have been reintroduced to treat infections caused by multidrug-resistant and extensively drug-resistant Gram-negative bacteria (Karaiskos et al., 2019).

Fosfomycin is a bactericidal antibiotic with a broad-spectrum antimicrobial activity against Gram-positive and Gram-negative microorganisms by inhibiting bacterial cell synthesis (Falagas et al., 2016; Sastry and Doi, 2016). In Gram-negative bacteria, resistance to fosfomycin can be mediated by mutations in the chromosomal fosfomycin transporter genes *uhpT* and *glpT*, amino acid modification or overexpression of the fosfomycin target MurA, and production of the fosfomycin-modifying enzymes such as FosA and FosC (Falagas et al., 2016; Sastry and Doi, 2016). Among them, glutathione S-transferase FosA encoded by chromosomes or plasmids is the main reason for widespread fosfomycin resistance in Gram-negative bacteria (Sastry and Doi, 2016; Ito et al., 2017).

To date, 10 *fosA* variants (*fosA1*∼*fosA10*) have been identified in *Enterobacteriaceae* since the first *fosA* (namely *fosA1*) described in *Serratia marcescens* in 1980 (Mendoza et al., 1980; Zurfluh et al., 2020). Fosfomycin resistance has been sporadically reported in *Salmonella*, one of the leading foodborne pathogens (Lin and Chen, 2015; Qi et al., 2019). So far, *fosA3*, *fosA4*, and *fosA7* have been detected in *Salmonella* (Ito et al., 2017; Rehman et al., 2017). FosA7, identified on the chromosomes of *Salmonella enterica* serovar Heidelberg from broiler chickens in Canada in 2017 (Rehman et al., 2017), has been detected globally in various *Salmonella* serotypes (e.g., Agona, Brandenburg, and Derby), *Escherichia coli*, and *Citrobacter koseri* from various sources (Supplementary Table 1). The *fosA7* gene has not yet been detected in *Salmonella* isolates from China. Thus, in this study, we aimed to investigate the prevalence of *fosA7* among *Salmonella* isolates from food animals, one pig slaughterhouse and retail meat products in China, and to evaluate the impact of *fosA7* on bacterial fitness.

## Materials and Methods

### Bacterial Strains, *fosA7* Detection, and Antimicrobial Susceptibility Testing

We obtained 264 *S. enterica* isolates including serovars London (*n* = 43), Kentucky (*n* = 35), Typhimurium (*n* = 34), Derby (*n* = 26), Rissen (*n* = 24), Indiana (*n* = 19), and others (*n* = 83) from 1,041 fecal samples from food-producing animals (pigs, chickens, and cattles) and 1,205 retail meat products (chicken meat, pork, and beef) in supermarkets or farmers’ markets in Gansu, Guangdong, Guizhou, Henan, Hubei, Jiangsu, Liaoning, Shandong, and Xinjiang provinces and Shanghai of China from July 2019 to June 2020 (Supplementary Table 2). Eighty-seven *Salmonella* isolates including serovars Typhimurium (*n* = 33), Rissen (*n* = 30), London (*n* = 14), Derby (*n* = 4), Reading (*n* = 4), and Pakistan (*n* = 2) were obtained from pig carcass swab samples and intestinal content samples in a slaughterhouse in Jiangsu province and nine *Salmonella* isolates including Wandsworth (*n* = 2), Lexington (*n* = 2), Reading (*n* = 1), Indiana (*n* = 1), Typhimurium (*n* = 1), London (*n* = 1), and Virchow (*n* = 1) from chicken meat from Chongqing, China, in 2016.

The presence of *fosA7* was detected by polymerase chain reaction and sequencing with the primers fosA7-F (5′-ACTTAACGCTTGCTGTC-3′) and fosA7-R (5′-TGCTAAATCTCCCACAT-3′). All *fosA7*-positive isolates were tested for their susceptibility to ampicillin, cefotaxime, meropenem, gentamicin, amikacin, streptomycin, tetracycline, chloramphenicol, florfenicol, nalidixic acid, ciprofloxacin, fosfomycin (with 25 mg/L glucose-6-phosphate), and sulfamethoxazole/trimethoprim using the agar dilution method and colistin using the microbroth dilution method. The results were interpreted according to CLSI M100, 30th edition (Clinical and Laboratory Standards Institute [CLSI], 2020). Streptomycin (≥32 mg/L) and florfenicol (≥ 32 mg/L) were interpreted according to EUCAST^1^ epidemiological cutoff values for *S. enterica*. Fosfomycin (> 32 mg/L) was interpreted according to EUCAST (see text footnote 1) breakpoint tables for *Enterobacterales*. *E. coli* American Type Culture Collection (ATCC) 25922 was used as quality control.

### Whole-Genome Sequencing and Analysis

The whole genomes of all *fosA7*-positive *Salmonella* were sequenced by Illumina Hiseq, and sequence reads were assembled into contigs with SPAdes v.3.13.0. The whole-genome sequences were analyzed for resistance genes, chromosome mutations, and plasmids using Center for Genomic Epidemiology pipelines^2^. Phylogenetic analysis of 30 *fosA7*-positive *S.* Derby isolates and ST71 *fosA7*-negative *S.* Derby 14C-S8N4 obtained from pork in Yangzhou, Jiangsu province, China, in 2014 based on 3002 cgMLST locus were performed using cgmlstfinder.py contained in the cgMLSTFinder service (Clausen et al., 2018). iTOL was used to make the phylogenetic tree visual (Letunic and Bork, 2021). Five *fosA7*-carrying *S*. Reading isolates in this study and *S*. Reading CVM N42528 (GenBank accession no. GCA_001481335.1) from pork chop in the United States were analyzed for the core genome regions by Parsnp software. The whole-genome sequencing data have been deposited in the GenBank under accession number PRJNA743999.

### In-Frame Deletion of Chromosomal *fosA7* in *S*. Derby

The *fosA7* deletion in *S.* Derby HA2-WA5 that exhibits susceptibility to all tested agents was performed by double exchange of homologous recombination as previously described using primers listed in Supplementary Table 3 (Guo et al., 2020). The deletion mutant HA2-WA5Δ*fosA7* was tested for minimum inhibitory concentration (MIC) of fosfomycin (25 mg/L glucose-6-phosphate).

### Cloning of *fosA7* and Functional Analysis

The *fosA7* of HA2-WA5 was amplified by polymerase chain reaction and cloned into the pBR322 vector. The vector pBR322 and the recombinant plasmid pBR322-*fosA7* were transformed into *E. coli* DH5α, *Salmonella* Typhimurium SL1344, *S.* Pullorum C79-13, *S*. Enteritidis P125109, *S.* Derby 14C-S8N4 (ST71, *fosA7*-negative), *Enterobacter cloacae* CMCC45301, and *Klebsiella pneumoniae* CMCC46117 *via* electroporation, selected by 32 mg/L ampicillin. The function of *fosA7* was determined by testing their MICs of fosfomycin (25 mg/L glucose-6-phosphate).

### Growth Curve and Plasmid Stability

The growth curves of all transformants, HA2-WA5 and HA2-WA5Δ*fosA7*, were determined in LB broth for 14 h at 37°C and 180 rpm. The overnight cultures were adjusted to an OD_600_ of 1 and then diluted 1:200 (10 μl/20 ml) in fresh LB broth. The OD_600_ of the cultures was measured every hour using Tecan Sunrise Microplate Reader (TECAN, Switzerland). Experiments were performed in triplicate.

To investigate the stability of pBR322 and pBR322-*fosA7* in different hosts, all transformants were maintained for 7 days in daily refreshed (100-fold dilution) LB broth without antibiotics. Cultures were streaked on LB agar plates, and 50 colonies were replica-plated on LB agar plates containing 32 mg/L ampicillin or 256 mg/L fosfomycin with 25 mg/L glucose-6-phosphate on each day.

### Growth Competition Experiments *in vitro*

To investigate the relative fitness (RF) of *fosA7*, *Salmonella* isolates SL1344-pBR322-*fosA7*, C79-13-pBR322-*fosA7*, 14C-S8N4-pBR322-*fosA7*, P125109-pBR322-*fosA7*, and HA2-WA5Δ*fosA7* were competed against SL1344-pBR322, C79-13-pBR322, 14C-S8N4-pBR322, P125109-pBR322, and HA2-WA5, respectively, in daily refreshed (1,000-fold dilution) LB broth for 96 h as previously described (Wu et al., 2018). At 48 and 96 h, the total number of bacteria was determined by plating proper diluted cultures on LB agar, and all colonies were replica-plated on LB agar with 16 mg/L fosfomycin and 25 mg/L glucose-6-phosphate. All competition experiments were performed at least three replicates. The RF was calculated as the percentage of transformants or deletion mutant in all isolates. An RF value > 0.5 indicated that the transformants/deletion mutant had a selective advantage over the original strain, whereas a score < 0.5 indicated a fitness cost.

### Statistical Analysis

The results were analyzed with GraphPad Prism version 7.0 (GraphPad Software, Inc., La Jolla, CA, United States).

## Results

### *Salmonella* Derby ST40 Was the Main Carrier for *fosA7*

Among 360 *Salmonella* isolates, *fosA7* was detected in 35 strains, with *S.* Derby (*n* = 30) being the dominant host. Five *S.* Reading were also positive for *fosA7*. The *fosA7*-carrying *Salmonella* isolates showed highest resistance to chloramphenicol (*n* = 31, 88.57%), followed by nalidixic acid (*n* = 30, 85.71%), tetracycline (*n* = 29, 82.86%), florfenicol (*n* = 27, 77.14%), sulfamethoxazole/trimethoprim (*n* = 27, 77.14%), and ampicillin (*n* = 27, 77.14%), but none of them were resistant to amikacin, meropenem, and colistin (Table 1). Notably, all *fosA7*-positive *Salmonella* isolates were still susceptible to fosfomycin, with MICs ranging between 1 and 32 mg/L (Table 1).

**TABLE 1:** Antimicrobial susceptibility results of *fosA7*-positive *Salmonella* isolates in this study.

Strains	Serovars	Source	Year	Fosfomycin MIC (mg/L)	Resistance phenotype
GZ19MPS2	Derby	Pork	2019	4	TET/NAL
GZ19MPS3	Derby	Pork	2019	2	TET/NAL/CIP
GZ19MPS17	Derby	Pork	2019	2	AMP/CTX/STR/TET/CHL/FFC/NAL/CIP/SXT
GZ19MPS20	Derby	Pork	2019	2	AMP/STR/TET/CHL/FFC/NAL/SXT
GZ19MPS26	Derby	Pork	2019	1	STR/TET/CHL/NAL/SXT
LN19FBS3	Derby	Cattle	2019	2	AMP/GEN/STR/CHL/FFC/NAL/CIP/SXT
LN19MPS11	Derby	Pork	2019	2	AMP/TET/CHL/NAL/CIP/SXT
LN19MPS18	Derby	Pork	2019	2	AMP/GEN/STR/TET/CHL/FFC/NAL/CIP/SXT
LN19MPS22	Derby	Pork	2019	2	AMP/GEN/STR/TET/CHL/FFC/NAL/CIP/SXT
SH19MPS13	Derby	Pork	2019	2	AMP/STR/TET/CHL/FFC/NAL/CIP/SXT
SH19MPS19	Derby	Pork	2019	2	TET/CHL/FFC/NAL/CIP/SXT
SH19MPS14	Derby	Pork	2019	1	AMP/STR/TET/CHL/FFC/NAL/SXT
SH19MPS23	Derby	Pork	2019	1	AMP/STR/TET/CHL/FFC/SXT
SH19MPS28	Derby	Pork	2019	2	AMP/STR/TET/CHL/FFC/NAL/CIP/SXT
SH19MPS29	Derby	Pork	2019	2	AMP/TET/CHL/FFC/NAL/SXT
XJ19BS1	Derby	Cattle	2019	1	STR/TET/NAL/SXT
YC19BS1	Derby	Cattle	2019	1	AMP/GEN/STR/CHL/FFC/NAL/CIP/SXT
YC19BS2	Derby	Cattle	2019	1	AMP/GEN/STR/CHL/FFC/NAL/CIP/SXT
YC19BS3	Derby	Cattle	2019	4	AMP/GEN/STR/CHL/FFC/NAL/CIP/SXT
YC19BS4	Derby	Cattle	2019	1	AMP/CTX/GEN/STR/TET/CHL/FFC/NAL/CIP/SXT
YC19BS5	Derby	Cattle	2019	1	AMP/GEN/STR/CHL/FFC/NAL/CIP/SXT
YZ19MPS46	Derby	Pork	2019	4	AMP/TET/CHL/FFC/SXT
YZ19MBS1	Derby	Beef	2019	2	AMP/STR/TET/CHL/FFC/NAL/SXT
WH19MPS1	Derby	Pork	2019	1	AMP/GEN/STR/TET/CHL/FFC/NAL/CIP/SXT
HN20MPS2	Derby	Pork	2020	1	AMP/STR/TET/CHL/FFC/NAL/CIP/SXT
SD20MPS5	Derby	Pork	2020	2	AMP/STR/TET/CHL/FFC/NAL/CIP/SXT
HA2-WA5	Derby	Pig slaughterhouse	2016	32	–
HA8-WA2	Derby	Pig slaughterhouse	2016	8	AMP/GEN/STR/TET/CHL/FFC/NAL/CIP/SXT
HA1-FLR3	Derby	Pig slaughterhouse	2016	8	AMP/GEN/STR/TET/CHL/FFC/NAL/CIP/SXT
HA9-EX2	Derby	Pig slaughterhouse	2016	32	AMP/GEN/TET/CHL/FFC/NAL/CIP/SXT
HA5-SE10	Reading	Pig slaughterhouse	2016	8	AMP/CTX/GEN/TET/CHL/FFC
HA3-FL1	Reading	Pig slaughterhouse	2016	16	AMP/CTX/GEN/TET/CHL/FFC
HA3-DE2	Reading	Pig slaughterhouse	2016	8	AMP/CTX/GEN/TET/CHL/FFC
HA7-LA4	Reading	Pig slaughterhouse	2016	32	TET/CHL/NAL
CQ2-72	Reading	Chicken meat	2016	16	TET/CHL/NAL

*– indicates that strain was susceptible to all tested antimicrobial agents.*

*AMP, ampicillin; CTX, cefotaxime; GEN, gentamicin; STR, streptomycin; TET, tetracycline; CHL, chloramphenicol; FFC, florfenicol; NAL, nalidixic acid; CIP, ciprofloxacin; SXT, sulfamethoxazole/trimethoprim.*

**FIGURE 1:**
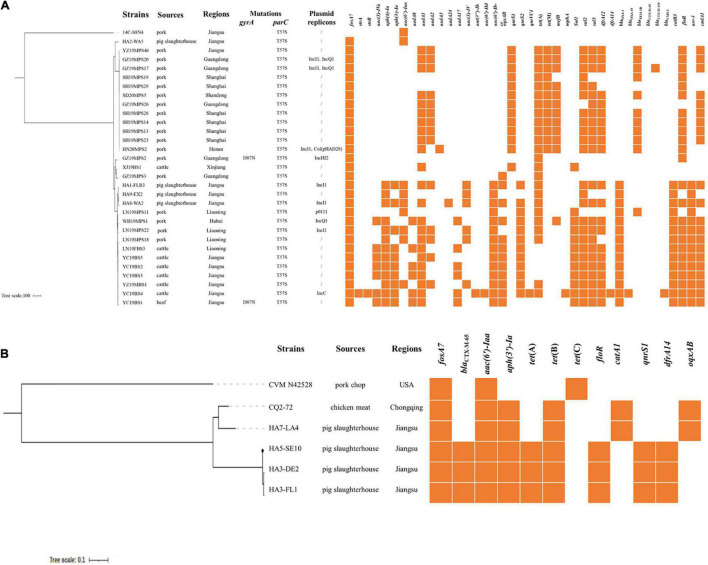
**(A)** Phylogenetic tree of *fosA7*-positive ST40 *S.* Derby compared with *fosA7*-negative ST71 *S*. Derby. Antibiotic resistance genes, mutations, and plasmid replicons are shown. **(B)** Phylogenetic tree of *fosA7*-positive *S*. Reading compared with *S*. Reading isolate CVM N42528 (GCA_001481335.1). Antibiotic resistance genes are shown. No mutations or plasmid replicons are identified.

Whole-genome sequencing showed that all *S.* Derby belonged to ST40, with one being ST40 variant (isolate YZ19MBS1), and five *S*. Reading strains were classified as ST1628. The *fosA7* gene was located on chromosomes of all *Salmonella* isolates in this study and differed from the firstly identified *fosA7* in *S.* Heidelberg by one (*S*. Reading) or 12 (*S*. Derby) nucleotide differences, resulting in one or five amino acid changes. Agreement with the first detected *fosA7* in *S.* Heidelberg ABB07-SB3031 (Rehman et al., 2017), the *fosA7* gene was surrounded by putative open reading frames. The genetic environment of *fosA7* and its neighboring region (∼24.3 kb) in all *S.* Derby and *S*. Reading strains obtained in this study were highly identical (> 98.0%) to the corresponding region of numerous *S.* Heidelberg strains, including ABB07-SB3031 (GAC_000973785) and *S*. Stanleyville strain CFSAN059881 (CP075116) (Supplementary Figure 1). In addition to *fosA7*, 35 *Salmonella* isolates carried one to 28 resistance genes, conferring resistance or decreasing susceptibility to β-lactams (*bla*_TEM_, *bla*_OXA_, *bla*_CTX–M_, and *bla*_CMY_), aminoglycosides [*aac(3)-IV*, *aac(3)-IVa*, *aph(4)-Ia*, *aph(3)-Ia*, *aac(6′)-Iaa*, *ant(3″)-Ih*, *aac(6′)-IId*, *aadA*, and *strAB*], quinolones [*qnrS*, *qnrVC*, *aac(6′)-Ib-cr*, and *oqxAB*], tetracyclines [*tet*(A), *tet*(B), and *tet*(M)], chloramphenicols (*catA1*, *catB3*, and *floR*), sulfonamides (*sul1*, *sul2*, and *sul3*), trimethoprims (*dfrA12* and *dfrA14*), macrolides (*mefB* and *mphA*), and rifampin (*arr-3*). Mutation within *parC* (T57S) was found in all *fosA7*-bearing *S.* Derby; *gyrA* (D87N) mutation was also observed in two of them (Figure 1A). No plasmid replicons and mutations in *gyrA* or *parC* were detected in *S*. Reading. Among the 30 *fosA7*-positive *S*. Derby, plasmid replicons were identified in 10 of them (Figure 1A).

### Phylogenetic Analysis of *fosA7*-Positive *S*. Derby and *S*. Reading

To further understand their genetic relationship, we performed a phylogenomic analysis on the *fosA7*-positive *S.* Derby and *S.* Reading. The results showed the division of 30 *fosA7*-positive *S.* Derby ST40 isolates into four clades (Figure 1A). Clade I only included one isolate, HA2-WA5, obtained from a pig slaughterhouse in 2016 with the least resistance genes. Twelve *S.* Derby isolates from pork were classified as clade II, and seven of them contained the same resistance genes. Clade III contained two isolates from pork and one isolate from cattle with few resistance genes. Fourteen isolates from various sources, including all *S.* Derby from cattle and beef, belonged to clade IV; numerous resistance genes were observed in this clade. Five *fosA7*-positive *S.* Reading were clustered in two clades (Figure 1B). The strains in the same clade had identical antimicrobial susceptibility profiles and resistance genes. The results indicate that clonal spread has occurred particularly in the same host.

### Deletion of *fosA7* Reduced Susceptibility of Fosfomycin and Slightly Affected Fitness

**FIGURE 2:**
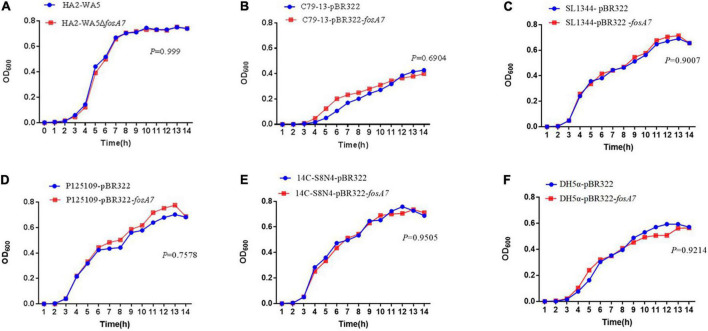
Growth curves of **(A)** *fosA7*-positive *S*. Derby HA2-WA5 and its *fosA7* deletion mutant; **(B)** *S*. Typhimurium SL1344-pBR322 and SL1344-pBR322-*fosA7*; **(C)** *S*. Pullorum C79-13-pBR322 and C79-13-pBR322-*fosA7*; **(D)** *S*. Enteritidis P125109-pBR322 and P125109-pBR322-*fosA7*; **(E)** *S*. Derby 14C-S8N4-pBR322 and 14C-S8N4-pBR322-*fosA7*; **(F)** *E. coli* DH5α-pBR322 and DH5α-pBR322-*fosA7*.

**FIGURE 3:**
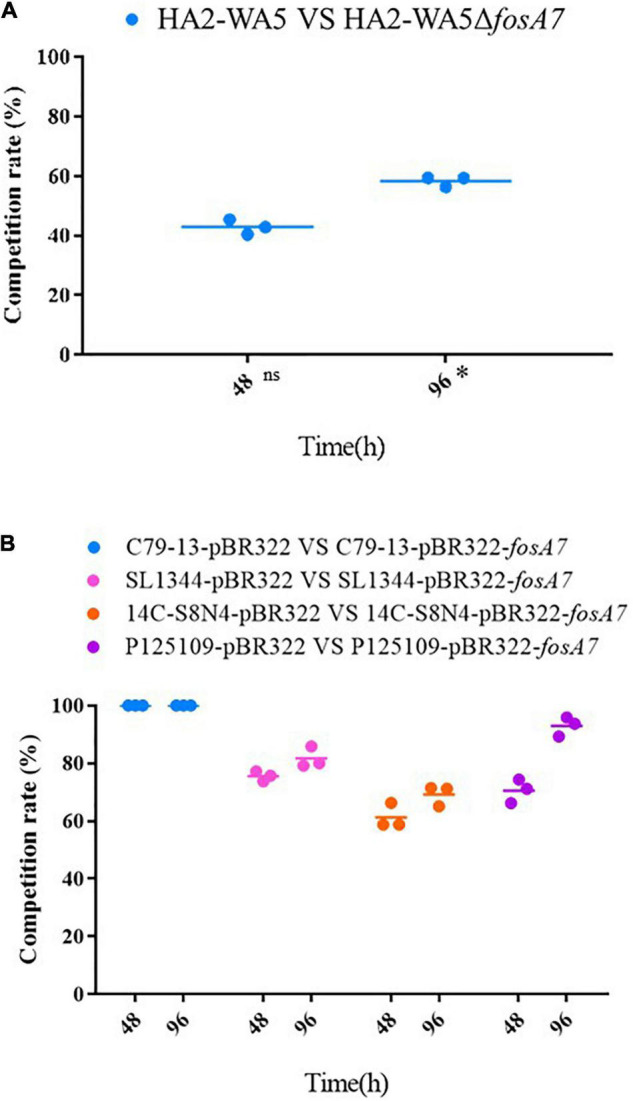
Effects of *fosA7* on bacterial relative fitness. **(A)** *fosA7*-positive *S*. Derby HA2-WA5 and its *fosA7* deletion mutant, ns indicates difference with no significance, * indicates difference with significance; **(B)** plasmid-borne *fosA7* in *S*. Pullorum C79-13, *S*. Typhimurium SL1344, *S*. Derby 14C-S8N4, and *S*. Enteritidis P125109. RF was calculated as percentage of transformants or deletion mutant in all isolate.

Because *fosA7* was located on the chromosome and *S.* Derby was the most common host, we constructed an in-frame *fosA7* deletion mutant to assess the impact of chromosomally encoded FosA7 in *S.* Derby. After deletion of *fosA7* in *S.* Derby HA2-WA5, its MIC of fosfomycin was reduced from 32 to 2 mg/L, further confirming the role of chromosomally located *fosA7* in reducing the susceptibility of fosfomycin. The growth curves of HA2-WA5 and HA2-WA5Δ*fosA7* were almost identical, suggesting that the *fosA7* deletion in the chromosome did not affect the growth (Figure 2A). Additionally, deletion of *fosA7* caused slightly decreased fitness at 48 h (*P* > 0.05) but enhanced fitness at 96 h with a biological advantage (0.01 < *P* < 0.05) (Figure 3A).

### Plasmid-Borne *fosA7* Significantly Increase Minimum Inhibitory Concentrations of Fosfomycin in *Salmonella*

Chromosomal-encoding FosA7 in *S*. Derby exhibited low MIC values to fosfomycin. To investigate whether the chromosomal *fosA7* in *S.* Derby could be mobilized to *fosA7*-negative bacteria and inactivated fosfomycin, we constructed recombinant plasmid pBR322-*fosA7* and transformed it to different hosts. The recombinant plasmid pBR322-*fosA7* was successfully transformed to *E. coli* DH5α, *S.* Typhimurium, *S.* Pullorum, *S. Enteritidis*, and *S.* Derby but failed to *K. pneumoniae* and *E. cloacae* after multiple electroporation experiments. Plasmid pBR322-*fosA7* was stable for 7 days in *S.* Typhimurium, *S.* Pullorum, *S.* Derby, and *E. coli* but was lost (∼4%) in *S.* Enteritidis on the last day of passage (Supplementary Figure 2).

The acquisition of *fosA7* significantly increased MICs of fosfomycin in *Salmonella* (1,024-fold) and conferred fosfomycin resistance, whereas *E. coli* DH5α was still susceptible after acquiring *fosA7* with 16-fold elevated MIC of fosfomycin (Supplementary Table 4).

### Plasmid-Borne *fosA7* Did Not Affect Bacterial Growth and Increased Fitness of *Salmonella*

To assess the impact of *fosA7* on the bacterial growth, growth curves of *E. coli*, *S.* Typhimurium, *S.* Pullorum, *S.* Enteritidis, and *S.* Derby carrying pBR322 or pBR322-*fosA7* were compared. The acquisition of *fosA7* rarely affected the growth of *E. coli* and *Salmonella* (Figures 2B–E).

The RF of *fosA7* was determined by growth competition *in vitro*; *E. coli* DH5α was not included because FosA7 did not confer fosfomycin resistance in DH5α. The *fosA7* gene significantly increased bacterial fitness, particularly in *S.* Pullorum (Figure 3B). *S.* Pullorum C79-13-pBR322-*fosA7* completely replaced C79-13-pBR322 at 48 h, showing a significant biological benefit. In *S.* Typhimurium, *fosA7* also exhibited fitness advantage (RF = 0.7555 ± 0.0168) at 48 h; no apparent difference was observed between 48 and 96 h (RF = 0.7920 ± 0.0710). Among four *Salmonella* serotypes, the least enhancement of fitness was shown in *S.* Derby (RF = 0.6924 ± 0.0435 at 96 h) after acquiring *fosA7*.

## Discussion

Since the identification of *fosA7* on the chromosomes of *S.* Heidelberg from broiler chickens in 2017, it has been detected in multiple *Salmonella* serotypes, with *S.* Heidelberg being the most common host (Supplementary Table 1). In this study, *fosA7* was detected in *S.* Derby and *S.* Reading among 27 serotypes, with *S.* Derby ST40 being predominant. In Europe, *S.* Derby has become the sixth most common human serotype and the most common in turkeys, as well as the second most frequently detected in pig carcasses and pigs in 2018 (European Food Safety Authority [EFSA], and European Centre for Disease Prevention and Control [ECDC], 2020). In China, *S*. Derby has also become one of the leading serovars associated with the pork production chain, with ST40 being predominant (Cai et al., 2016; Jiu et al., 2020). ST40, mainly isolated from the pork sector, is the dominant ST type within *S*. Derby; however, *S*. Derby isolates from the poultry mainly belong to ST71, particularly originating from Europe (Sévellec et al., 2019). The *fosA7* gene is located on the chromosomes of all tested *S*. Derby isolates belonging to ST40; the global spread of *S*. Derby ST40, particularly in pigs and pork, will facilitate the worldwide dissemination of *fosA7*.

Genes on the chromosomes of various species could act as reservoirs of potential resistance genes; they have the opportunity to be captured by mobile genetic elements (e.g., insertion sequences, transposons, plasmids, and integrative conjugative elements) and to become mobilized resistance genes to horizontally disseminate between different bacteria (Partridge, 2011). For instance, *fosA8* may be mobilized from the chromosome of natural fosfomycin-resistant enterobacterial species *Leclercia adecarboxylata* to IncN plasmid in *E. coli*, thus conferring high-level fosfomycin resistance in *E. coli* (Poirel et al., 2019). IncN plasmids carrying *fosA8* further spread in *E. coli* and *K. pneumoniae* and cointegrated into IncFII or IncR plasmid, respectively (Biggel et al., 2021). Also, the most popular plasmid-mediated fosfomycin resistance gene *fosA3* was likely originated from *Kluyvera georgiana via* IS*26*-mediated capture and further jumped into various types of plasmids such as IncHI2 and F33:A-: B-, thus rapidly disseminating in *Enterobacteriaceae* (Ito et al., 2018; Fang et al., 2020; Lv et al., 2020a). It seems that *fosA7* is exclusively chromosomal in *S*. Derby and *S*. Reading, which accords with previously reported *S*. Heidelberg (Rehman et al., 2017). Analysis of the genetic structure of *fosA7* suggests that it may exist as an intrinsic gene being the progenitor/origin of the *fosA7* gene and may have other functions, which need further investigation. Although all *fosA7*-positive *Salmonella* isolates in this study were still susceptible to fosfomycin, *fosA7* can confer high-level fosfomycin resistance in *Salmonella* if transferred on plasmids. It is possible due to the expression difference of FosA7 encoding by multicopy plasmid instead of the chromosome. The precise reason will be further investigated. Interestingly, a similar phenomenon was reported in *K. georgiana* (Ito et al., 2018). As the possible origin of plasmid-mediated *fosA3*, chromosomally located *fosA* in *K. georgiana* strains YDC799 and ATCC 51603 exhibited fosfomycin MICs of 32 and 0.5 mg/L, respectively, but showed high-level resistance to fosfomycin (MIC > 1,024 mg/L) for *E. coli* after cloning into a plasmid vector (Ito et al., 2018). Thus, although chromosome-located *fosA7* only reduces the susceptibility of fosfomycin in *Salmonella*, it can allow the bacterium to survive under selective pressure, e.g., low level of fosfomycin in the environment. On the other hand, it has the potential to be a reservoir of a mobilized fosfomycin resistance gene. The emergence of *fosA7* with a transposase gene downstream on the chromosome of *E. coli* or *fosA7.5* flanked by insertion sequences located on plasmid further supports this hypothesis (Milner et al., 2020; Pan et al., 2021). The *fosA7* gene can confer high-level fosfomycin resistance once it is captured by a high copy plasmid or the mobile elements that bring stronger promoters that enhance its expression.

However, *E. coli* DH5α remained susceptible after the acquisition of pBR322-*fosA7*, showing a 16-fold elevated MIC of fosfomycin. Similarly, the intrinsic chromosomal *fosA* of *Pseudomonas aeruginosa* contributed to a 16-fold increase of fosfomycin MIC in *E. coli* TOP10 (Ito et al., 2017). A novel FosA7.5 variant was identified and conferred fosfomycin resistance in *E. coli*. It differed from FosA7 in *Salmonella* by four amino-acid substitutions, whereas three FosA7.5 variants (one or two amino-acid changes) in *E. coli* resulted in fosfomycin resistance or susceptibility (Milner et al., 2020). It indicates the critical role of key amino acid differences in FosA7 in fosfomycin resistance within different hosts, which may also explain susceptibility to fosfomycin in *fosA7*-positive *S*. Derby and *S*. Reading in this study.

Generally, antibiotic resistance is associated with a fitness cost that is usually observed as a reduction in bacterial growth rate or competitive ability, such as colistin resistance gene *mcr-1* (Andersson and Hughes, 2010; Yang et al., 2017). However, the fitness cost caused by resistance could be alleviated by compensatory mutations (Björkman and Andersson, 2000). Fitness cost and its compensation are two key factors that determine the spread and stability of antibiotic resistance in bacteria (Björkman and Andersson, 2000). For example, the plasmid-encoded efflux pump TMexCD1-TOprJ1 confers resistance to tetracyclines and reduces susceptibility to cephalosporins, aminoglycosides, and quinolones, barely affecting the growth of *K. pneumoniae*, but significantly reducing the growth of *E. coli* and *S*. Typhimurium (Lv et al., 2020b). It may explain why *tmexCD1-toprJ1* is mainly described in *K. pneumoniae* (Lv et al., 2020b; Sun et al., 2020; Hirabayashi et al., 2021). In this study, it appears that the deletion of chromosomally located *fosA7* slightly affects the fitness of *S*. Derby ST40, yet the acquisition of plasmid-borne *fosA7* not only confers high-level fosfomycin resistance but also obviously improves the bacterial fitness of *Salmonella*. It may facilitate the dissemination and persistence of *fosA7* in bacterial populations.

To conclude, *fosA7* will widely disseminate with the spread of specific *Salmonella* serovars, such as *S*. Derby ST40, in our study. Although chromosomally borne *fosA7* in *S*. Derby and *S*. Reading only reduce susceptibility to fosfomycin, it will help the organisms to survive under low-level of fosfomycin and has the potential to be a reservoir of the mobilized fosfomycin resistance gene. Because it can induce high-level resistance to fosfomycin and enhance bacterial fitness in *Salmonella* if transferred on plasmids, and plasmid-mediated *fosA7.5* has already been detected in *E. coli*, it needs further surveillance.

## Data Availability Statement

The datasets presented in this study can be found in online repositories. The names of the repository/repositories and accession number(s) can be found below: https://www.ncbi.nlm.nih.gov/bioproject/PRJNA743999/.

## Author Contributions

JW and XJ conceived the study. YW, HW, JW, C-YM, and P-CS carried out the experiments. JW, YW, and Z-YW analyzed the data. JW wrote the manuscript. Z-MP and XJ revised the manuscript. All authors read and approved the final manuscript.

## Conflict of Interest

The authors declare that the research was conducted in the absence of any commercial or financial relationships that could be construed as a potential conflict of interest.

## Publisher’s Note

All claims expressed in this article are solely those of the authors and do not necessarily represent those of their affiliated organizations, or those of the publisher, the editors and the reviewers. Any product that may be evaluated in this article, or claim that may be made by its manufacturer, is not guaranteed or endorsed by the publisher.

## Footnotes

1
http://www.eucast.org

2
http://www.genomicepidemiology.org/
