# Supplementary material for: Chromosomally Located fosA7 in Salmonella Isolates From China
Source: Front Microbiol. 2021 Dec 28;12:781306. doi: 10.3389/fmicb.2021.781306 (PMC8751274; doi:10.3389/fmicb.2021.781306)
Supplement: Supplementary file 1 [file Data_Sheet_1.pdf]

Table S1. Characteristics of *fosA7* in Enterobacteriaceae from animals, food, human, and the environment

Gene	Species	*fosA7* location	Country	sources	Reference
fosA7	*S*. Heidelberg	Chromosome	Canada	chicken	Rehman et al., 2017
fosA7	*S*. Heidelberg	Chromosome	USA	ground Turkey, human	Keefer et al., 2019
fosA7	*S*. Heidelberg, S. Derby	-	USA	dog	Cummings et al.,2020
fosA7	*S*. Derby	-	Brazil	food, patient	de Melo et al., 2021
fosA7	*S*. Infantis	-	Serbia	poultry farm	Jovčić et al., 2020
fosA7	*S*. Agone	-	USA	papaya	Pightling et al., 2020
fosA7	*S*. Grumpensis, S. Saphra	-	Brazil	food (viscera, chicken meat, boneless chicken thigh)	Monte et al., 2021
fosA7	*S*. Brandenburg, *S*. Heidelberg	-		Food (chicken breast, chicken wing), chicken cage after cleaning	Monte et al., 2019
fosA7	*C. koseri*	-	South Africa	sewage sludge	Ekwanzala et al., 2020
fosA7	-	-	USA	environment, clinical	Hua et al., 2020
fosA7.5	*E. coli*	plasmid	Canada	patient	Milner et al., 2020
fosA7	*E. coli*	chromosome	China	cattle, sheep, patients	Pan et al., 2021

-, not available.

Cummings, K.J., Mitchell, P.K., Rodriguez-Rivera, L.D., and Goodman, L.B. (2020). Sequence analysis of *Salmonella enterica* isolates obtained from shelter dogs throughout Texas. *Vet. Med. Sci.* 6: 975-979. doi: 10.1002/vms3.320

de Melo, A.N.F., Monte, D.F.M., de Souza Pedrosa, G.T., Balkey, M., Jin, Q., Brown, E., et al. (2021). Genomic investigation of antimicrobial resistance determinants and virulence factors in *Salmonella enterica* serovars isolated from contaminated food and human stool samples in Brazil. *Int. J. Food Microbiol.* 343, 109091. doi: 10.1016/j.ijfoodmicro.2021.109091

Ekwanzala, M.D., Dewar, J.B., Kamika, I., and Momba, M.N.B. 2020. Genome sequence of carbapenem-resistant *Citrobacter koseri* carrying $bla_{\text{OXA-181}}$ isolated from sewage sludge. *J. Glob. Antimicrob. Resist.* 20, 94-97. doi: 10.1016/j.jgar.2019.07.011

Hua M, Huang W, Chen A, Rehmet M, Jin C, and Huang Z. 2020. Comparison of antimicrobial resistance detected in environmental and clinical isolates from historical data for the US. *Biomed. Res. Int.* 2020:4254530. doi: 10.1155/2020/4254530

Jovčić, B., Novović, K., Filipić, B., Velhner, M., Todorović, D., Matović, K., *et al*. 2020. Genomic characteristics of colistin-resistant *Salmonella enterica* subsp. *enterica* serovar Infantis from poultry farms in the Republic of Serbia[J]. *Antibiotics*. 9, 886. doi: 10.3390/antibiotics9120886

Keefer, A.B., Xiaoli, L., M'ikanatha, N.M., Yao, K., Hoffmann, M., et al. (2019). Retrospective whole-genome sequencing analysis distinguished PFGE and drug-resistance-matched retail meat and clinical *Salmonella* isolates. *Microbiology.* 165, 270-286. doi: 10.1099/mic.0.000768

Milner, K.A., Bay, D.C., Alexander, D., Walkty, A., Karlowsky, J.A., Mulvey, M.R., et al. (2020). Identification and characterization of a novel FosA7 member from fosfomycin-resistant *Escherichia coli* clinical isolates from Canadian hospitals. *Antimicrob. Agents Chemother*. 65, e00865-20. doi: 10.1128/AAC.00865-20

Monte, D.F., Lincopan, N., Berman, H., Cerdeira, L., Keelara, S., Thakur, S., et al. 2019. Genomic features of high-priority *Salmonella enterica* serovars circulating in the food production chain, Brazil, 2000-2016. *Sci. Rep.* 9,11058. doi: 10.1038/s41598-019-45838-0

Monte, D.F.M., Nethery, M.A., Barrangou, R., Landgraf, M., and Fedorka-Cray, P.J. 2021. Whole-genome sequencing analysis and CRISPR genotyping of rare antibiotic-resistant *Salmonella enterica* serovars isolated from food and related sources. *Food Microbiol*. 93, 103601. doi: 10.1016/j.fm.2020.103601

Pan, Y., Hu, B., Bai, X., Yang, X., Cao, L., Liu, Q., et al. (2021). Antimicrobial resistance of non-O157 shiga toxin-producing *Escherichia coli* isolated from humans and domestic animals. *Antibiotics*. 10, 74. doi: 10.3390/antibiotics10010074

Pightling, A.W., Pettengill, J., Luo, Y., Strain, E., and Rand, H. 2020. Genomic diversity of *Salmonella enterica* isolated from papaya samples collected during multiple outbreaks in 2017. *Microbiology*. 166, 453-459. doi: 10.1099/mic.0.000895

Rehman, M.A., Yin, X., Persaud-Lachhman, M.G., and Diarra, M.S. (2017). First detection of a fosfomycin resistance gene, *fosA7*, in *Salmonella enterica* serovar Heidelberg isolated from broiler chickens. *Antimicrob. Agents Chemother*. 61, 410-417. doi: 10.1128/AAC.00410-17

Table S2. ***Salmonella enterica*** **isolates obtained from July 2019 to June 2020 used in this study**

Serovars	Sources (no.)					Total
	pig	cattle	chicken meat	pork	beef	
Albany			2			2
Anatum			1	4		5
Braenderup			9	1		10
Corvallis			3			3
Derby		7		18	1	26
Enteritidis			9			9
Give			1	1		2
Goldcoast				7		7
Indiana			19			19
Infantis				1		1
Kentucky	1		33	1		35
Kottbus			2			2
Litchfield				6		6
Livingstone				4		4
London	4		3	20	16	43
Mbandaka		1				1
Newport				10		10
Putten			2			2
Rissen	2		2	19	1	24
Saintpaul			2	7		9
Senftenberg				1		1
Thompson			2			2
Typhimurium	4		1	28	1	34
Uganda				7		7

Table S3. Primers used to delete ***fosA7*** **in** ***S.*** **Derby HA2-WA5**

Primer name	Primer sequence (5 to 3)	Size (bp)
fosA7-UpF	GAGCGGATAACAATTTGTGGAATCCCGGGAAGCTGATGATATGCTTTTTCGCAAG	436
fosA7-UpR	CTAAATCTCCTGCAAATTACTGACAGCAAGCGTTA	
fosA7-DownF	GTAATTTGCAGGAGATTTAGCATCCCGACTGACGC	377
fosA7-DownR	AGCGGAGTGTATATCAAGCTTATCGATACCTGTACATTCAAGACATTATTGCAGA	
fosA7-InF	TGTTGAGTGGGATACTGGCGCTTAC	229
fosA7-InR	CGGATCAAGAAAATAGAAAGATTGC	
fosA7-OutF	AGCTGATGATATGCTTTTTCGCAAG	1571
fosA7-OutR	TCTCGTCAGCGATGTCTTCCT	(1270)

Table S4. MICs of fosfomycin against parental strains and pBR322-*fosA7* transformations

Strains	Fosfomycin MIC (mg/L)
E. coli DH5α	0.25
DH5α-pBR322	0.25
DH5α-pBR322-*fosA7*	4
S. Typhimurium SL1344	0.5
SL1344-pUC57	0.5
SL1344-pUC57-tetA-variant	512
S. Pullorum C79-13	0.5
C79-13-pBR322	0.5
C79-13-pBR322-*fosA7*	512
S. Enteritidis P125109	0.5
P125109-pBR322	0.5
P125109-pBR322-*fosA7*	512
S. Derby 14C-S8N4	0.5
14C-S8N4-pBR322	0.5
14C-S8N4	512

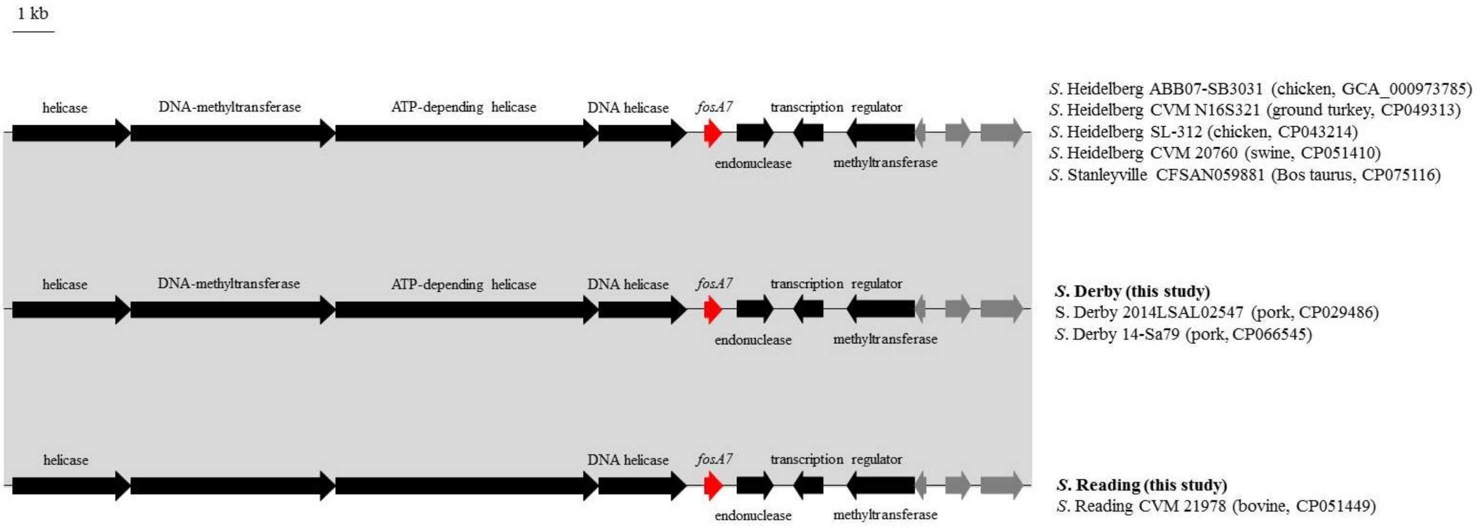

Figure S1. Genetic environment of *fosA7* and its neighboring region (~24.3 kb) in *Salmonella* Derby and *Salmonella* Reading isolates in this study and comparison with other *fosA7*-carrying regions in *Salmonella*. Regions with > 98% homology are shaded in gray.

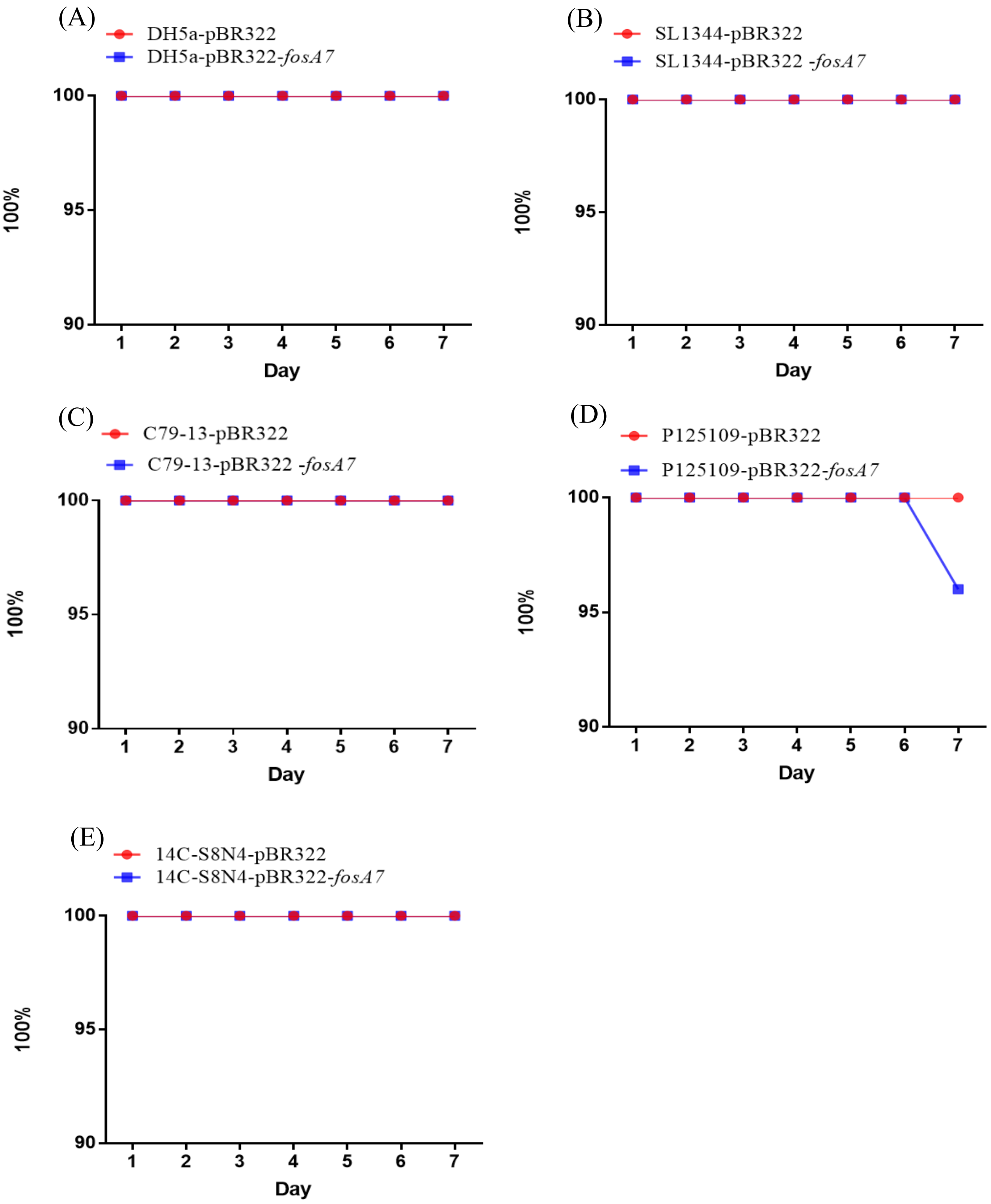

Figure S2. Plasmid stability of pBR322 and pBR322-*fosA7* in *E. coli* and *Salmonella*. (A) *E. coli* DH5α; (B) *S.* Typhimurium SL1344; (C) *S.* Pullorum C79-13; (D) *S.* Enteritidis P125109; (E) *S.* Derby 14C-S8N4.
